# Use of Single-Injection Recombinant Vesicular Stomatitis Virus Vaccine to Protect Nonhuman Primates Against Lethal Nipah Virus Disease

**DOI:** 10.3201/eid2506.181620

**Published:** 2019-06

**Authors:** Chad E. Mire, Joan B. Geisbert, Krystle N. Agans, Krista M. Versteeg, Daniel J. Deer, Benjamin A. Satterfield, Karla A. Fenton, Thomas W. Geisbert

**Affiliations:** Galveston National Laboratory, Galveston, Texas, USA; University of Texas Medical Branch, Galveston

**Keywords:** Nipah virus, Henipavirus, vaccines, nonhuman primates, viruses, zoonoses, vesicular stomatitis virus

## Abstract

Nipah virus (NiV) is a zoonotic pathogen that causes high case-fatality rates (CFRs) in humans. Two NiV strains have caused outbreaks: the Malaysia strain (NiV_M_), discovered in 1998–1999 in Malaysia and Singapore (≈40% CFR); and the Bangladesh strain (NiV_B_), discovered in Bangladesh and India in 2001 (≈80% CFR). Recently, NiV_B_ in African green monkeys resulted in a more severe and lethal disease than NiV_M_. No NiV vaccines or treatments are licensed for human use. We assessed replication-restricted single-injection recombinant vesicular stomatitis vaccine NiV vaccine vectors expressing the NiV glycoproteins against NiV_B_ challenge in African green monkeys. All vaccinated animals survived to the study endpoint without signs of NiV disease; all showed development of NiV F Ig, NiV G IgG, or both, as well as neutralizing antibody titers. These data show protective efficacy against a stringent and relevant NiV_B_ model of human infection.

Nipah virus (NiV) and Hendra virus (HeV) are highly pathogenic zoonotic agents in the paramyxovirus genus *Henipavirus*. Human case-fatality rates (CFRs) for these viruses historically have ranged from 40% to >90% (*1*). NiV is categorized as a Biosafety Level 4 (BSL-4) pathogen because of the substantial illness and death it causes and the lack of approved vaccines and therapeutic drugs for human use. In 2015, the World Health Organization listed NiV as a priority pathogen because it is likely to cause severe outbreaks and, in early 2018, placed NiV on the Blueprint list of priority diseases (https://www.who.int/blueprint/priority-diseases). This WHO designation was bolstered because of a deadly NiV outbreak (CFR 89%) during spring 2018 in southwestern India, where NiV had not previously been reported (*2*).

Bats of the genus *Pteropus* are the primary reservoir in nature for NiV (*3*), but several other mammal species can be infected by NiV (*4*–*7*). Analysis of NiV genomes has identified 2 NiV strains responsible for outbreaks: Malaysia strain NiV_M_ and Bangladesh strain (NiV_B_). NiV_M_ caused the first identified outbreak of NiV during 1998–1999 in Malaysia and Singapore (≈270 persons infected; CFR ≈40%) (*8*,*9*) and perhaps was responsible for a 2014 outbreak in the Philippines (CFR ≈52%); however, this speculation is based on short genomic reads, so the NiV strain that caused this outbreak is not known (*10*). NiV_B_ has caused repeated outbreaks in Bangladesh and northeastern India; outbreaks occurred almost every year during 2001–2015 (*11*–*15*). These NiV_B_ outbreaks had higher CFRs, averaging ≈80% (*14*), and showed documented human-to-human transmission (*11*,*16*).

Eight experimental preventive candidate vaccines against henipaviruses have been evaluated in NiV_M_ animal models: 1) canarypox and 2) vaccinia viruses encoding the NiV_M_ fusion protein (F) or the NiV_M_ attachment protein (G) that have shown protection against NiV_M_ in hamsters and pigs (*17*,*18*); 3) a recombinant adeno-associated vaccine expressing the NiV_M_ G protein that completely protected hamsters against homologous NiV_M_ challenge (*19*); 4) recombinant vesicular stomatitis viruses (rVSVs) expressing the NiV_M_ F protein or the NiV_M_ G protein that had 100% efficacy in hamsters against NiV_M_ (*20*); 5) rVSVs expressing the NiV_B_ F protein or the NiV_B_ G protein that completely protected ferrets from NiV_M_ disease (*21*); 6) an rVSV expressing the Zaire ebolavirus (EBOV) glycoprotein (GP) and the NiV_M_ G protein (rVSV-EBOV-GP-NiVG) that demonstrated efficacy in NiV_M_ hamster (*22*) and African green monkey (*Chlorocebus aethiops*) (*23*) models; 7) a recombinant measles virus vector expressing the NiV_M_ G protein that had efficacy in the NiV_M_ African green monkey model (*24*); and 8) a recombinant subunit vaccine based on the HeV G protein (sG_HeV_) that completely protected small animals against lethal HeV and NiV_M_ infections (*25*–*27*) and was efficacious in the robust African green monkey model of HeV (*28*) and NiV_M_ infection (*29*). Of 8 vaccines, the sG_HeV_ vaccine is furthest along in evaluation; it has received licensure as a veterinary vaccine for HeV in horses (Equivac HeV, Zoetis, https://www.zoetis.com) in Australia and is being considered as a human vaccine against NiV. When tested against NiV, these 8 vaccine vectors have been tested only against NiV_M_ infection in animal models, and although the antigenicity of these vaccines should not be a concern given that HeV G is an immunogen against NiV_M_ infection, there are new data on the NiV_B_ African green monkey model to consider as far as dose/regimen of vaccines. 

NiV_B_ infection in African green monkeys is more pathogenic than NiV_M_ infection (*30*). This difference resulted in significantly reduced efficacy of antibody therapy because of temporal differences in viral load. Specifically, the human monoclonal antibody m102.4 that had been shown to completely protect African green monkeys against lethal NiV_M_ disease when treatment was delayed until day 5 after virus exposure provided no protection when African green monkeys were challenged with NiV_B_ and treated beginning at day 5 after virus challenge (*30*,*31*). Considering these new data, the current vaccines against NiV need to be evaluated for possible differences in dose/regimen against the more pathogenic NiV_B_ infection in the robust African green monkey model. To assess single-dose vaccine efficacy, we evaluated the rVSV vaccine vectors expressing either the NiV_B_ F or NiV_B_ G proteins 28 days after a single-dose vaccination in the NiV_B_ African green monkey model, which most faithfully recapitulates human disease (*5*,*30*).

## Methods

### rVSV Vaccine Vectors and NiV_B_ Challenge Stock

We recovered the rVSV NiV_B_ vaccines (rVSV-ΔG-NiV_B_/F-GFP and rVSV-ΔG-NiV_B_/G-GFP) and rVSV-ΔG-GFP using methods as previously described (*21*,*32*). The isolate of NiV_B_ used in this vaccine study was obtained from a fatal human case (200401066) described previously (*30*).

### Statistical Analyses

**Figure 1:**
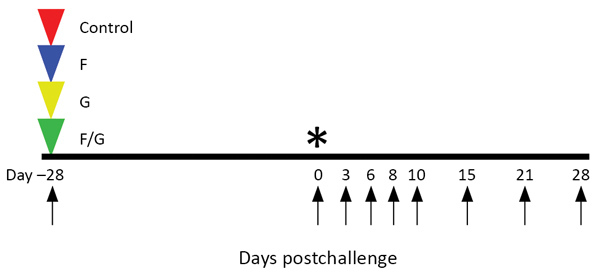
Groups of African green monkeys (*Chlorocebus aethiops*) receiving recombinant vesicular stomatitis virus (rVSV) vaccine against Nipah virus Bangladesh strain (NiV_B_). Triangles indicate days of vaccination; arrows indicate days of sampling; and asterisk (*) indicates day of challenge. Red indicates control group (G_Ind_*rVSV-ΔG-GFP expressing no glycoprotein); blue indicates F group (G_Ind_* rVSV-NiV_B_/F-GFP expressing the NiV_B_ F protein); yellow indicates G group (G_Ind_*rVSV-NiV_B_ /G-GFP expressing the NiV_B_ G protein); F/G group (single-cycle infectious virions with NiV_B_ F and G proteins on the cell surface). F, fusion; G, attachment

Animal studies in BSL-4 and nonhuman primate work generally restrict the number of animals used, the volume of biological samples that can be obtained, and the ability to repeat assays independently and thus limit statistical analysis. Consequently, we present these data as the mean calculated from replicate samples, not replicate assays, and error bars represent SD across replicates (Figure 1, panels B, C, D).

### Animal Ethics Considerations and Experiments

Healthy adult African green monkeys were handled in the animal BSL-4 containment space at the Galveston National Laboratory (Galveston, TX, USA). Research was approved under animal protocol 1310040 by the University of Texas Medical Branch Institutional Animal Care and Use Committee (Appendix).

We used 10 adult African green monkeys weighing 3.5–6.0 kg in this study. One animal served as control (received G_In_* rVSV-ΔG-GFP), and 3 animals per vaccine group received G* rVSV-ΔG-NiV_B_/F-GFP, G* rVSV-ΔG-NiV_B_/G-GFP, or rVSVΔG-NiV_B_/F/G. For vaccination, animals were anesthetized with ketamine and vaccinated with ≈10^7^ PFU by intramuscular injection (day −28). Twenty-eight days after vaccination, the animals were exposed to ≈5 × 10^5^ PFU of NiV_B_; the dose was equally divided between the intratracheal and the intranasal routes for each animal. Animals were monitored for clinical signs of illness (i.e., temperature, respiration quality, blood count, and clinical pathologic findings) at 0, 3, 6, 8, 10, 15, 21, and 28 days postchallenge (dpc).

### NiV_B_ Serum Neutralization Assays

We determined neutralization titers against NiV_B_ using a conventional serum neutralization assay. In brief, we serially diluted serum 5-fold or 2-fold depending on magnitude of neutralization titers and incubated with ≈100 PFU of NiV_B_ for 1 h at 37°C, as previously described (*30*).

### RNA Isolation from NiV_B_-Infected African green monkeys

We isolated RNA from NiV_B_-infected animals as described previously (*30*). For viremia, we added 100 μL of blood to 600 μL of AVL viral lysis buffer (QIAGEN, https://www.qiagen.com) for RNA extraction. For virus load in tissue, we stored ≈100 mg in 1 mL RNAlater (QIAGEN) for 7 d to stabilize RNA, removed the RNAlater completely, and homogenized tissues in 600 μL RLT buffer (QIAGEN) in a 2-mL cryovial using a tissue lyser (QIAGEN) and ceramic beads.

### Detection of NiV_B_ Load

We isolated RNA from blood or tissues and assessed it using primers and probe targeting the N gene and the intergenic region between N and P genes of NiV_B_ for quantitative reverse transcription PCR (qRT-PCR). The probe used was 6FAM-5′CGT CAC ACA TCA GCT CTG ACA A 3′-6TAMRA (Life Technologies, https://www.thermofisher.com), as described previously (*30*).

### Hematology and Serum Biochemistry

We assessed clinical pathology of NiV_B_-infected African green monkeys by hematology and serum biochemistry analysis as described previously (*30*). We performed the hematology assays using a laser-based hematologic analyzer (Beckman Coulter, https://www.beckmancoulter.com) and serum biochemistry analysis using a Piccolo point-of-care analyzer and Biochemistry Panel Plus analyzer discs (Abaxis, https://www.abaxis.com). 

### Histopathology and Immunohistochemistry

We performed necropsies on all animals and collected tissue samples of all major organs. We performed histopathologic and immunohistochemical examination and analyses as described previously (*30*).

## Results

### Immunization of African Green Monkeys and Measuring the Humoral Immune Response

**Figure 2:**
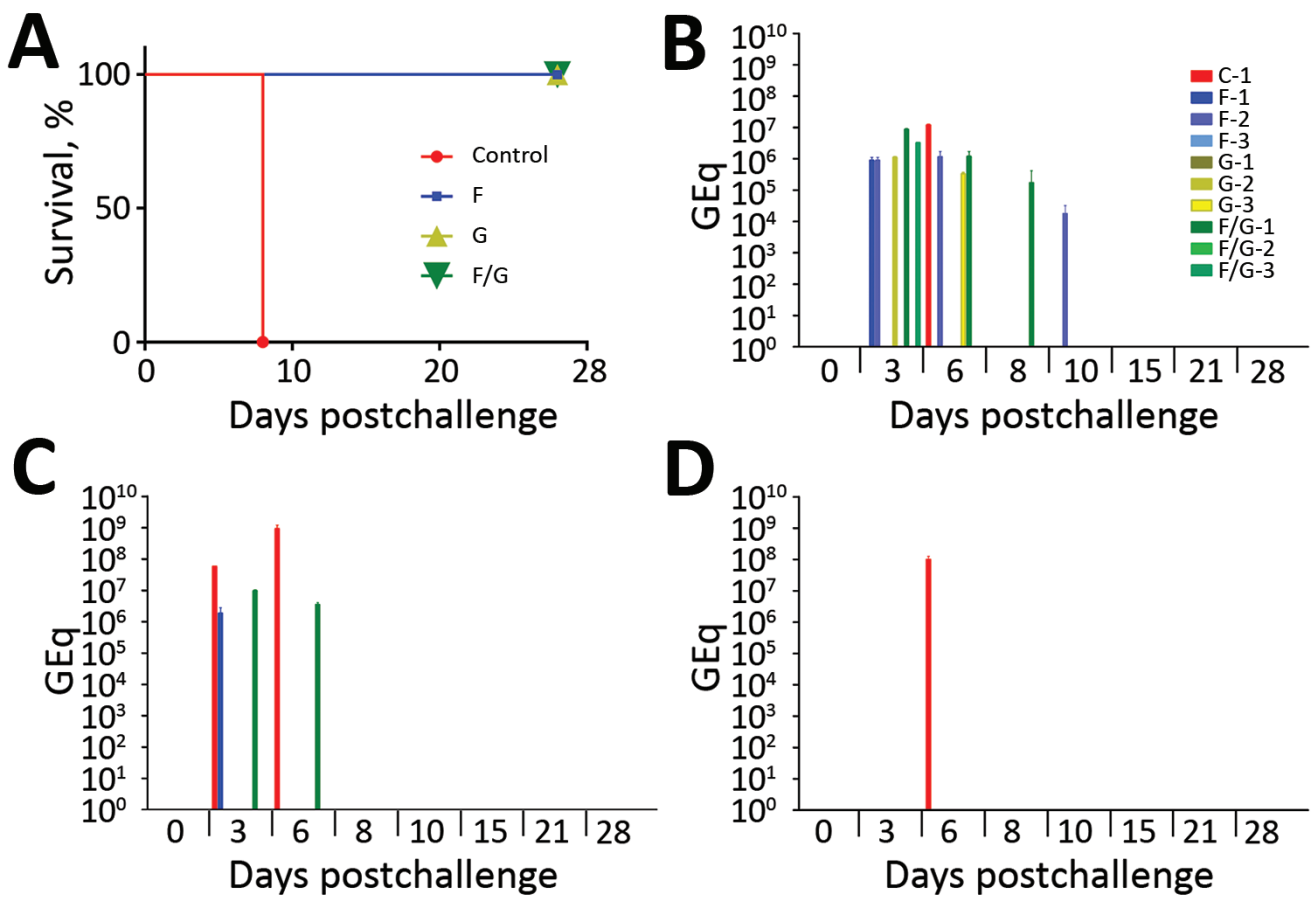
Protection of African green monkeys (*Chlorocebus aethiops*) from Nipah virus Bangladesh strain (NiV_B_)–mediated disease and viral load. A) Kaplan-Meier survival curve for each vaccine group and historical controls after NiV_B_ challenge: controls (vaccine, n = 1; historical, n = 14), F group (n = 3), G group (n = 3), and F/G group (n = 3). C–D) Viral load in the animals as detected by NiV_B_ GEq by reverse transcription quantitative PCR from nasal swab samples: as GEq per swab (B), oral swab samples as GEq per swab (C), and blood as GEq/mL (D). Red, control group (G_Ind_*rVSV-ΔG-GFP expressing no glycoprotein); blue, F group (G_Ind_* rVSV-NiV_B_ /F-GFP expressing the NiV_B_ F protein); yellow, G group (G_Ind_*rVSV-NiV_B_/G-GFP expressing the NiV_B_ G protein); green, F/G group (single-cycle infectious virions with NiV_B_ F and G proteins on the cell surface). Error bars indicate SD. C, control; F, fusion; G, attachment; GEq, genome equivalent.

Previously, single-injection, single-round replication rVSV vaccine vectors expressing the NiV_B_ F or NiV_B_ G proteins were described, characterized, and shown to be efficacious against NiV_B_ challenge in ferrets (*21*). To assess the efficacy of these vectors in the NiV_B_ African green monkey model, 4 groups of African green monkeys received a single intramuscular vaccination of rVSV vectors on day −28 (Figure 2). To analyze the antibody response to rVSV-ΔG-NiV_B_ vaccinations, we assessed circulating antibodies for neutralization activity against NiV_B_ before and after vaccination by using a 50% plaque-reduction neutralization titer (PRNT_50_) assay. All 4 groups had no detectable neutralizing antibody titers before vaccination (Table 1, day −28). On the day of challenge, the control animal (C-1) did not have detectable neutralizing antibody titers against NiV_B_, whereas all animals from the specific NiV protein vaccination groups (F, G, and F/G) had detectable neutralizing antibodies against NiV_B_ (Table 1, day 0). Overall, the detectable neutralizing antibody response against NiV_B_ reached a 1:640 dilution titer in the G and F/G groups and from 1:160 to 1:640 in the F group.

**Table 1:** NiV_B_ Serum neutralization titers in vaccinated African green monkeys (*Chlorocebus aethiops*)*

Vaccine	Animal no.	Day −28†	Day 0†	Day 28†
None	C-1	<20	<20	40‡
F only vaccine	F-1	<20	640	1,280
	F-2	<20	160	2,560
	F-3	<20	320	5,120
G only vaccine	G-1	<20	640	5,120
	G-2	<20	640	5,120
	G-3	<20	640	5,120
F+G vaccine	F/G-1	<20	640	2,560
	F/G-2	<20	640	5,120
	F/G-3	<20	640	2,560

*Titers are reciprocal serum dilution at which 50% of virus was neutralized. NiV, Nipah virus; NiV_B_, NiV Bangladesh strain. †Day postchallenge. ‡Terminal day 8 postchallenge.

### NiV_B_ Challenge and Viral Load of Vaccinated African Green Monkeys

To determine the efficacy of the rVSV-ΔG-NiV_B_ vectors against NiV_B_ disease in African green monkeys, we challenged these animals by combined intratracheal and intranasal routes with a lethal challenge dose of NiV_B_ on day 0 (Figure 1). All African green monkeys were closely monitored for up to 28 dpc for clinical signs of illness. The NiV_B_ antigen vaccinated animals in the F (F-1–3), G (G-1–3), and F/G (F/G-1–3) groups showed no signs of clinical illness (Table 2) and were 100% protected against NiV_B_ challenge (Figure 1, panel A), whereas the animal in the nonspecific vaccinated control group (C-1) exhibited clinical signs of disease (Table 2) and died of infection on day 8 (Figure 1, panel A). In addition, the control animal was the only NiV_B_-infected animal to have lymphopenia and serosanguinous nasal discharge during the course of disease (Table 2).

**Table 2:** Clinical findings and outcome of Nipah virus Bangladesh strain–challenged African green monkeys*

Animal no.	Sex	Group	Clinical illness	Clinical and gross pathology findings†
C-1	F	Control ΔG vaccine	Loss of appetite (d 6–8); labored breathing (d 6–8). Died on d 8.	Lymphopenia (d 6); serosanguinous nasal and oral discharge (d 8), serosanguinous pleural fluid, severely inflated, enlarged lungs with severe congestion and hemorrhage of all lobes, multifocal to coalescing hemorrhage of the mucosal surface of the urinary bladder.
F-1	F	F vaccine	None	Thrombocytopenia (d 15); >3 fold increase in ALT (d 6), >3 fold increase in AST
F-2	M	F vaccine	None	None
F-3	M	F vaccine	None	Increase in CRP (d 6)
G-1	F	G vaccine	None	None
G-2	M	G vaccine	None	None
G-3	M	G vaccine	None	None
F/G-1	F	F + G vaccine	None	Increase in CRP (d 8)
F/G-2	M	F + G vaccine	None	Thrombocytopenia (d 21, d 28); increase in CRP (d 8, d 10, d 15)
F/G-3	M	F + G vaccine	None	Thrombocytopenia (d 8)

*ALT, alanine aminotransferase; AST, aspartate aminotransferase; CRP, C-reactive protein. †Lymphopenia is defined as a >30% decrease in number of lymphocytes; thrombocytopenia is defined as a >30% decrease in number of platelets.

To determine the level of NiV_B_ replication in animals after challenge, we assessed viral load by qRT-PCR on nasal and oral swab samples and whole blood samples (Figure 1, panels B–D). We detected NiV_B_ genome equivalents (GEq) from nasal swab samples (Figure 1, panel B) in the control, F, G, and F/G groups. The following animals were positive for viral RNA: C-1 at 6 dpc; F-1 at 3 dpc; F-2 at 3, 6, and 10 dpc; G-1 at 3 dpc; G-3 at 6 dpc; F/G-1 at 3, 6, and 8 dpc; and F/G-3 at 3 dpc. At 6 dpc, when C-1 was positive for NiV RNA in nasal swab samples, the levels were >1 log higher than they were for the NiV-antigen vaccinated groups F, G, and F/G. Oral swab samples were negative for NiV RNA in all animals in the G-vaccinated group (Figure 1, panel C), and NiV_B_ GEq were detected from oral swab samples in the control, F, and F/G groups. The following animals were positive for viral RNA: C-1 at 3 and 6 dpc, F-1 at 3 dpc, and F/G-1 at 3 and 6 dpc. Within these oral swab sample results, C-1 had NiV RNA levels up to 100-fold higher than the F and F/G animals that had positive oral swab samples (Figure 1, panel C). Unlike the results for swab samples, which represent tissues initially exposed to NiV, systemic and circulating NiV_B_ GEq were not detected in whole blood from animals in the F, G, and F/G groups, whereas the control animal was positive in the blood sample from 6 dpc (Figure 1, panel D). The lack of systemic and circulating detection of NiV_B_ RNA correlated with survival (Table 2; Figure 1, panel A).

### Gross Pathologic, Histopathologic, and Immunohistochemical Analyses of NiV_b_-Infected African Green Monkeys

In the F, G, and F/G groups, we observed no gross pathologic findings at study endpoint. However, in the control animal that died of NiV_B_ infection, gross pathologic findings included serosanguinous pleural effusion, failure of all lung lobes to collapse with severe pulmonary hemorrhage and congestion, and multifocal to coalescing hemorrhage of the mucosal surface of the urinary bladder.

**Figure 3:**
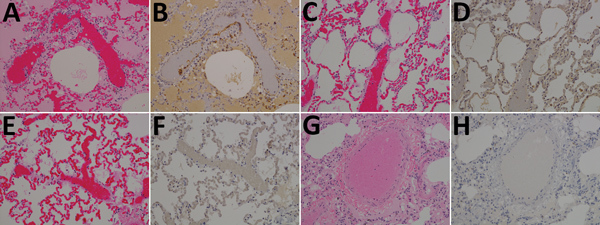
Results of testing for Nipah virus (NiV) in lung tissue from representative vaccinated African green monkeys (*Chlorocebus aethiops*). A, C, E, G) Hematoxylin and eosin staining; B, D, F, H) immunohistochemistry of tissues labeled with NiV N protein–specific polyclonal rabbit antibody. In stained tissue from the control animal (A), diffuse thickening of alveolar septae by moderate numbers of lymphocytes, plasma cells, polymerized fibrin, and edema fluid within the alveolar spaces were found; stained sections examined from the NiV F (C), NiV G (E), and NiV F/G (G) groups were unremarkable in comparison with sections from the control animal. In antibody-labeled tissue from the control animal (B), strong immunolabeling for NiV antigen with alveolar septae, scattered alveolar macrophages, and the endothelium of small caliber vessels were found, including syncytial cells with strong cytoplasmic immunolabeling for NiV antigen; no immunolabeling for NiV antigen was identified from the NiV F (D), NiV G (F), and NiV F/G (H) groups. Original magnification ×20.

Lung sections examined from the control animal had moderate lymphoplasmacytic interstitial pneumonia characterized by a diffuse thickening of alveolar septae by moderate numbers of lymphocytes, plasma cells, polymerized fibrin, and edema fluid. The alveolar spaces were flooded by edema fluid, polymerized fibrin, foamy alveolar macrophages, and cellular debris. Endothelial syncytial cells were most apparent in medium- to small-caliber vessels (Figure 3, panel A). The animals in the F, G, and F/G groups had no major histologic findings in the lung sections (Figure 3, panels C, E, G). Immunohistochemical analysis revealed strong NiV antigen immunoreactivity within scattered alveolar macrophages and the endothelium of the alveolar septae and syncytial cells within medium to small caliber vessels in up to ≈75% of the examined pulmonary tissues (Figure 3, panel B). The lung sections of the F, G, and F/G groups were devoid of detectable NiV antigen (Figure 3, panels D, F, H).

**Figure 4:**
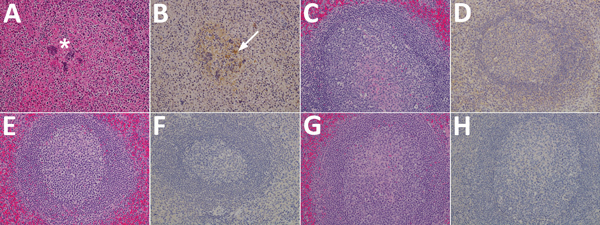
Results of testing for Nipah virus (NiV) in spleen tissue from representative vaccinated African green monkeys (*Chlorocebus aethiops*). A, C, E, G) Hematoxylin and eosin staining; B, D, F, H) immunohistochemistry of tissues labeled with NiV N protein–specific polyclonal rabbit antibody. In stained tissue from the control animal (A), moderate necrosis and drop out of the white pulp (*), with hemorrhage, and fibrin within germinal centers are seen; stained sections examined from the NiV F (C), NiV G (E), and NiV F/G (G) groups were devoid of any significant lesions compared with sections from the control animal. In antibody-labeled tissues from the control animal (B), strong immunolabeling for NiV antigen with scattered mononuclear cells (white arrow) and syncytial cells within germinal centers were found, and the endothelium of small caliber vessels had strong cytoplasmic immunolabeling for NiV antigen; no immunolabeling for NiV antigen was identified from the NiV F (D), NiV G (F), and NiV F/G (H) groups. Original magnification ×20.

Spleen sections from the control animal were depleted of lymphocytes in the multifocal follicular germinal centers within the splenic white pulp and were effaced by hemorrhage, fibrin, syncytial cell formation (Figure 4, panel A). Spleens from the F, G, and F/G groups had no major histologic findings (Figure 4, panels C, E, G). Immunohistochemical analysis of the spleen from the control animal revealed strong immunoreactivity for NiV antigen within the endothelium, syncytial cells, and scattered mononuclear cells in up to ≈50% of the examined splenic tissue (Figure 4, panel B), whereas the spleen sections of groups F, G, and F/G were devoid of detectable NiV antigen (Figure 4, panels D, F, H).

## Discussion

An important step in the preclinical development of a vaccine is efficacy testing in standards of animal models of disease. For NiV, the standard is the African green monkey model. Although the initial studies on the NiV_M_ model in African green monkeys were reported as near uniformly lethal, data from several groups have revealed the model is not 100% lethal, depending on dose and route of infection (*5*,*24*,*29*–*31*,*33*,*34*). Combining the control animals from these studies, in which African green monkeys were challenged with various combinations of routes (e.g., intratracheal, intranasal, intraperitoneal, oral, small particle aerosol) at various doses, revealed that 18 (72%) of 25 animals died; however, most of the control animals were positive for circulating NiV RNA and had signs of clinical disease to varying degrees. Historically, our previous studies with the NiV_B_ model has resulted in the deaths of all 14 control African green monkeys; the mean time to death was 7.14 days (Figure 2, panel A). We recently compared the pathogenesis of NiV_M_ and NiV_B_ strains in African green monkeys and observed that NiV_B_ caused more pulmonary and splenic pathologic findings (*30*). We also observed the efficacy of time to treatment post-NiV challenge with a human monoclonal antibody m102.4 was shorter for NiV_B_-infected animals than for NiV_M_-infected animals (*30*). With these animal data in mind and the fact that NiV_B_ has been responsible for most NiV outbreaks since 2002, we wanted to test our rVSV NiV vaccine vectors expressing NiV_B_ F and G proteins as immunogens, which had 100% efficacy against NiV_M_ challenge in ferrets (*21*), against NiV_B_ challenge in African green monkeys.

In this study, we vaccinated 1 control African green monkey with a nonglycoprotein rVSV vector control, G_Ind_* rVSV-ΔG-GFP, and 3 groups of 3 African green monkeys with NiV antigen vectors: G_Ind_* rVSV-ΔG-NiV_B_/F-GFP, G_Ind_* rVSV-ΔG-NiV_B_/G-GFP, or G_Ind_* rVSV-ΔG-NiV_B_/F/G-GFP. The control animal, C-1, did not develop NiV_B_ neutralizing antibodies by the day of challenge; had detectable circulating NiV RNA at 6 dpc; had clinical signs of NiV-mediated disease; and ultimately died of infection, showing typical NiV gross pathology and histopathologic findings. Conversely, the 3 rVSV NiV vaccine groups had animals in which detectable circulating NiV F, G, or F and G IgG developed, and circulating neutralizing antibody titers developed in all 3 groups by 28 days postvaccination. Each vaccine cohort had detectable NiV_B_ RNA in nasal swab samples and only the F and F/G groups in oral swab samples, but none of the cohorts had any detectable circulating NiV_B_ RNA throughout the course of the study. Consistent with the vaccine response from each cohort and the control of systemic spread of NiV_B_ infection and control of NiV-mediated disease, all of the specifically vaccinated African green monkeys survived NiV_B_ challenge.

The results of this study are similar to what we observed with these rVSV NiV constructs in the ferret model, which showed 100% protection regardless of the vaccine construct (*21*). Differences were that we found higher PRNT_50_ results for neutralizing antibody titers on day of challenge in this study and detected no circulating NiV RNA in the African green monkeys but did have detectable viral RNA at 6 dpc in the ferret study. Although we did not detect circulating viral RNA in the African green monkeys, the increase of neutralizing antibody titers at the study endpoint suggests sterilizing immunity was not achieved, and dosing or regimen will require further testing to reach sterilizing immunity with this single-round replication vaccine vector.

The single-round replication rVSV NiV vectors in this study and the replication competent rVSV-EBOV-GP-NiVG (*23*) are the only vaccine vectors to show 100% single-dose vaccine efficacy against NiV in the African green monkey model. Although both studies used this model, they differed in several ways. Our study used NiV_B_ and challenged through the intratracheal and intranasal routes, whereas the other study used NiV_M_ by the intratracheal route only (intratracheal challenge route used in initial model [*5*]). Here, we report detectable levels of NiV RNA in nasal swab samples at early times postchallenge, whereas the rVSV-EBOV-GP-NiVG study did not report any detectable NiV RNA in nasal swab samples. Whether these differences resulted from use of the intranasal route as part of the challenge cannot be determined here; however, neither study reported circulating levels of NiV RNA, indicating the prevention of systemic spread of NiV infection. Both studies reported the detection of circulating neutralizing antibodies on the day of challenge (28 [this study] and 29 days postvaccination). However, we reported on PFU reduction, and the rVSV-EBOV-GP-NiVG study reported on reduction of 200 50% tissue culture infectious dose in a tissue culture infectious dose assay, so the peak neutralizing titers at NiV challenge cannot be directly compared. 

The PRNT_50_ titers we reported can be directly compared with the recombinant subunit sG_HeV_ vaccine NiV study in African green monkeys that also was 100% efficacious (*29*), whereas we detected higher PRNT_50_ titers against NiV from the single injection of single-round replication vectors (from 160 to 640; Table 1) versus the PRNT_50_ titers 2 weeks after boost vaccination (from 28 to 379) for the recombinant subunit sG_HeV_ vaccine. However, these lower titers most likely are due to the sG_HeV_ vaccine being heterotypic because the PRNT_50_ titers against HeV in a similar African green monkey study were 640–1,280 on day of challenge (*28*). The development of neutralizing antibodies to the NiV glycoproteins after vaccination are important for protection, as highlighted by a single monoclonal antibody against the henipavirus G protein, m102.4, that is 100% protective against HeV, NiV_M_, and NiV_B_ when administered at least 3 dpc (*30*,*31*,*35*). 

In our study, the F cohort did not produce as consistent a neutralizing antibody titer response as did the G and F/G cohorts. Further analysis also revealed that, although no major changes occurred in hematologic and blood chemistry results for any of the vaccine cohorts, minor changes occurred in the F and F/G cohorts (Table 1). These data, taken together with the lack of detectable NiV_B_ RNA in the oral swab samples of the G group, suggest the rVSV NiV G vector might be the better option among the 3 vaccine vectors.

In summary, we found that single-round replication rVSV vectors against NiV_B_ provided 100% efficacy against NiV_B_ challenge using a single-dose regimen. The rVSV vaccine platform has received attention recently because the replication-competent rVSV-ZEBOV GP vaccine vector against EBOV has now been given to >16,000 humans in clinical trials ranging from phase 1 to phase 3 and has been safe and efficacious (*36*); however, data for pregnant women and immunocompromised persons are not yet available. A single-round replication rVSV vaccine vector that is immunogenic and efficacious would have an attractive safety profile. Whether these single-round replication rVSV NiV vaccine vectors are as safe as the recombinant subunit sG_HeV_ vaccine has yet to be determined, and the subunit vaccine has yet to be tested with a single-dose vaccine regimen. Although multidose vaccine regimens would be a potential strategy for laboratory and healthcare workers and for first responders in stable settings with defined risk for an NiV outbreak, an outbreak setting or a case of deliberate release of NiV would require rapid protection with a single administration of vaccine. The single-dose strategy was successfully enacted using a close-contact ring vaccination strategy with the rVSV-ZEBOV-GP vaccine at the end of the 2013–2016 EBOV epidemic (*37*–*39*). The strategy was so successful that it became the World Health Organization recommendation for future EBOV outbreaks and has recently been set into motion in the ongoing outbreak in the Democratic Republic of the Congo (*40*). Recent studies also suggest that the ring vaccination strategy for viruses such as EBOV (depending on transmissibility) that are endemic to countries that might not be able to afford a mass herd-immunity vaccination strategy might be more effective than mass vaccinations at controlling outbreaks (*41*). Further studies should examine the time to immunity of the G_Ind_* rVSV-ΔG-NiV_B_/G in the NiV_B_ African green monkey model because these data will be instrumental in providing information about whether this vaccine vector could be implemented in a ring vaccination strategy during future NiV outbreaks, such as the current one in India (*2*).

Appendix Additional methods for study on use of single-injection recombinant vesicular stomatitis virus vaccine to protect nonhuman primates against lethal Nipah virus disease.
