# Supplementary material for: Use of Single-Injection Recombinant Vesicular Stomatitis Virus Vaccine to Protect Nonhuman Primates Against Lethal Nipah Virus Disease
Source: Emerg Infect Dis. 2019 Jun;25(6):1144–52. doi: 10.3201/eid2506.181620 (PMC6537706; doi:10.3201/eid2506.181620)
Supplement: Appendix — Additional methods for study on use of single-injection recombinant vesicular stomatitis virus vaccine to protect nonhuman primates against lethal Nipah virus disease. [file 18-1620-Techapp-s1.pdf]

Article DOI: https://doi.org/10.3201/eid2506.181620

Use of Single-Injection Recombinant Vesicular Stomatitis Virus Vaccine to Protect Nonhuman Primates Against Lethal Nipah Virus Disease

Appendix

Additional Methods

rVSV Vaccine Vectors and NiV_B Challenge Stock

The rVSV NiV_B vaccines (rVSV-ΔG-NiV_B/F-GFP and rVSV-ΔG-NiV_B/G-GFP) and rVSV-ΔG-GFP were recovered using methods as previously described (*1*,*2*). These rVSVΔG viruses were propagated on BHK-21 cells transiently expressing the VSV glycoprotein (G_{Ind}) and were titered as previously described (*2*). Viruses complemented with VSV G_{Ind} are denoted as G_{Ind}* rVSV-ΔG-GFP, G*rVSV-ΔG-NiV_B/F-GFP, and G* rVSV-ΔG-NiV_B/G-GFP. To make the vaccine in the F/G group, as previously described Vero 76 cells were co-infected with G*rVSV-ΔG-NiV_B/F-GFP and G* rVSV-ΔG-NiV_B/G-GFP at MOI 5 for each virus (*1*). Supernatants were collected 24 h post infection (h.p.i.) and titered on BHK-21 cells transiently expressing VSV G_{Ind}.

The isolate of NiV_B used in this vaccine study was an isolate obtained from a fatal human case, 200401066 (kindly provided by Dr. Thomas G Ksiazek), as described previously (*3*).

Statistics

Animal studies in BSL-4 and NHP work generally restricts the number of animal subjects, the volume of biologic samples that can be obtained, and the ability to repeat assays independently and thus limit statistical analysis. Consequently, data are presented as the mean calculated from replicate samples, not replicate assays, and error bars represent the SD across replicates.

Animal Ethics and Experiments

Healthy, adult African green monkeys (AGMs) were handled in the animal BSL-4 containment space at the Galveston National Laboratory (GNL), Galveston, Texas. Research was approved under animal protocol 1310040 by the University of Texas Medical Branch (UTMB) Institutional Animal Care and Use Committee (IACUC). Animal research at UTMB was conducted in compliance with the Animal Welfare Act and adhered to the guidelines in the eighth edition of the Guide for the Care and Use of Laboratory Animals (*4*). This facility is fully accredited by the Association for Assessment and Accreditation of Laboratory Animal Care International. All steps were taken to ameliorate the welfare and to avoid the suffering of the animals in accordance with the "Weatherall report for the use of nonhuman primates" recommendations (royalsociety.org/-/media/Royal_Society_Content/policy/publications/2006/Weatherall-Report.pdf). Animals were housed in adjoining individual primate cages allowing social interactions, under controlled conditions of humidity, temperature, and light (12-h light/12-h dark cycles). Food and water were available ad libitum. Animals were monitored (pre- and post-infection) and fed commercial monkey chow, treats, and fruit twice daily by trained personnel. Environmental enrichment consisted of commercial toys. All procedures were conducted by trained personnel under the oversight of an attending veterinarian, and all invasive clinical procedures were performed while animals were anesthetized using ketamine or telazol. Endpoint criteria was specified and approved by the UTMB IACUC. Animals were euthanized using a pentobarbital-based euthanasia solution.

Ten adult AGMs weighing 3.5–6.0 kg were used in this studyb with 1 AGM control (received G_{Ind}* rVSV-ΔG-GFP) and 3 AGMs per vaccine group (received either G*rVSV-ΔG-NiV_B/F-GFP, G* rVSV-ΔG-NiV_B/G-GFP, or rVSVΔG-NiV_B/F/G). For vaccination, animals were anesthetized with ketamine and vaccinated with $\approx 10^7$ plaque forming units (PFU) by i.m. injection on day −28. Twenty-eight days post-vaccination, AGMs were exposed to $\approx 5 \times 10^5$ PFU of NiV_B with the dose being equally divided between the i.t. and the intranasal i.n. routes for each animal. After challenge, animals were monitored for clinical signs of illness, including temperature, respiration quality, blood count, and clinical pathology on days 0, 3, 6, 8, 10, 15, 21, and 28 post-challenge.

NiV_B Serum Neutralization Assays

Neutralization titers against NiV_B were determined by a conventional serum neutralization assay. Briefly, sera were serially diluted 5-fold or 2-fold, respectively, and incubated with ≈100 PFU of NiV_B for 1 h at 37°C as done previously (*3*). Virus and antibodies mixtures were then added Vero cells, and after 48 h plates were stained with neutral red and plaque forming units were counted 24 h after staining. The 50% neutralization titer was determined as the serum dilution at which at there was a 50% reduction in plaque counts versus control wells.

RNA Isolation from NiV_B-Infected AGMs

On procedure days, 100 μL of blood was added to 600 μL of AVL viral lysis buffer (Qiagen) for RNA extraction. For tissues, ≈100 mg was stored in 1 mL RNAlater (Qiagen) for 7 d to stabilize RNA. RNAlater was completely removed, and tissues were homogenized in 600 μL RLT buffer (Qiagen) in a 2-mL cryovial using a tissue lyser (Qiagen) and ceramic beads. The tissues sampled included conjunctiva, tonsil, oro/nasopharynx, nasal mucosa, trachea, right bronchus, left bronchus, right lung upper lobe, right lung middle lobe, right lung lower lobe, right lung upper lobe, right lung middle lobe, right lung lower lobe, bronchial lymph node (LN), heart, liver, spleen, kidney, adrenal gland, pancreas, jejunum, colon transversum, brachial plexus, brain (frontal and cerebellum), brain stem, cervical spinal cord, pituitary gland, mandibular LN, salivary gland LN, inguinal LN, axillary LN, mesenteric LN, urinary bladder, testes or ovaries, and femoral bone marrow. All blood samples were inactivated in AVL viral lysis buffer, and tissue samples were homogenized and inactivated in RLT buffer before removal from the BSL-4 laboratory. Subsequently, RNA was isolated from blood and nasal and oral swabs using the QIAamp viral RNA kit (Qiagen), and from tissues using the RNeasy minikit (Qiagen) according to the manufacturer’s instructions supplied with each kit.

Detection of NiV_B Load

RNA was isolated from blood or tissues and assessed using primers/probe targeting the N gene and intergenic region between N and P of NiV_B for reverse transcription quantitative PCR (RT-qPCR) with the probe used here being 6FAM-5′CGT CAC ACA TCA GCT CTG ACA A 3′-6TAMRA (Life Technologies, Carlsbad, CA, USA) as described previously (*3*). This strategy uses the intergenic region which only allows for genome and anti-genome detection without

detecting contaminating viral mRNA. NiV_B RNA was detected using the CFX96 detection system (Bio-Rad) in One-step probe RT-qPCR kits (Qiagen) with the following cycle conditions: 50°C for 10 min, 95°C for 10 sec, and 40 cycles of 95°C for 10 sec and 59°C for 30 sec. Cycle threshold (C_t) values representing NiV_B genomes were analyzed with CFX Manager Software, and data are presented as GEq. To generate the GEq standard curve, RNA from NiV_B challenge stocks was extracted, and the number of genomes was calculated using Avogadro's number and the molecular weight of the NiV_B genome.

Hematology and Serum Biochemistry

Prior to the study, baseline blood and sera were collected from all 10 AGMs on days −28, 0, 3, 6, 8, 10, 15, 21, and 28 post-challenge for analysis. Hematologic analysis including total leukocyte counts, leukocyte differentials, red blood cell counts, platelet counts, hematocrit values, total hemoglobin concentrations, mean cell volumes, mean corpuscular volumes, and mean corpuscular hemoglobin concentrations were analyzed from blood collected in tubes containing EDTA using a laser-based hematologic analyzer (Beckman Coulter). Serum samples were tested for concentrations of albumin, amylase, alanine aminotransferase (ALT), aspartate aminotransferase (AST), alkaline phosphatase (ALP), γ-glutamyltransferase (GGT), glucose, cholesterol, total protein, total bilirubin (TBIL), blood urea nitrogen (BUN), creatine (CRE), and C-reactive protein (CRP) by using a Piccolo point-of-care analyzer and Biochemistry Panel Plus analyzer discs (Abaxis).

Histopathology and Immunohistochemistry

Necropsy was performed on all subjects. Tissue samples of all major organs were collected for histopathologic and immunohistochemical examination and were immersion-fixed in 10% neutral buffered formalin for at least 21 d in BSL-4. After 21 d, the formalin was changed out; specimens were removed from BSL-4, processed in BSL-2 and embedded in paraffin and sectioned at 5 μm thickness. For immunohistochemistry, specific anti-NiV immunoreactivity was detected using an anti-NiV N protein rabbit primary antibody at a 1:5000 dilution for 30 min. The tissue sections were processed for immunohistochemistry using the Dako Autostainer (Dako). Secondary antibody used was biotinylated goat anti-rabbit IgG (Vector Laboratories, Burlingame, CA, USA) at 1:200 for 30 min followed by Dako LSAB2 streptavidin-HRP (Dako) for 15 min. Slides were developed with Dako DAB chromagen (Dako)

for 5 min and counterstained with hematoxylin for 1 min. Non-immune rabbit IgG was used as a negative staining control.

References

1. Mire CE, Versteeg KM, Cross RW, Agans KN, Fenton KA, Whitt MA, et al. Single injection recombinant vesicular stomatitis virus vaccines protect ferrets against lethal Nipah virus disease. Virol J. 2013;10:353. PubMed http://dx.doi.org/10.1186/1743-422X-10-353

2. Whitt MA, Geisbert TW, Mire CE. Single-vector, single-injection recombinant vesicular stomatitis virus vaccines against high-containment Viruses. Methods Mol Biol. 2016;1403:295–311. PubMed http://dx.doi.org/10.1007/978-1-4939-3387-7_16

3. Mire CE, Satterfield BA, Geisbert JB, Agans KN, Borisevich V, Yan L, et al. Pathogenic Differences between Nipah Virus Bangladesh and Malaysia strains in primates: implications for antibody therapy. Sci Rep. 2016;6:30916. PubMed http://dx.doi.org/10.1038/srep30916

4. National Research Council. Guide for the Care and Use of Laboratory Animals. Washington, DC: National Academies Press; 2013. https://www.ncbi.nlm.nih.gov/books/NBK54050/
